# Incidence and Predictors of Calf Morbidity and Mortality From Birth to 6-Months of Age in Dairy Farms of Northwestern Ethiopia

**DOI:** 10.3389/fvets.2022.859401

**Published:** 2022-05-23

**Authors:** Yeshwas F. Alemu, Wudu T. Jemberu, Zeleke Mekuriaw, Reta Duguma Abdi

**Affiliations:** ^1^School of Animal Science and Veterinary Medicine, Bahir Dar University, Bahir Dar, Ethiopia; ^2^International Livestock Research Institute, Addis Ababa, Ethiopia; ^3^College of Veterinary Medicine and Animal Sciences, University of Gondar, Gondar, Ethiopia; ^4^College of Veterinary Medicine, Long Island University, Brookville, NY, United States

**Keywords:** Bahir Dar milk-shed, calf morbidity, calf mortality, dairy farms, survival analysis

## Abstract

The Ethiopian government has initiatives for expanding the commercial and smallholder market-oriented urban and peri-urban dairy production systems to meet the demands for dairy products. However, there have been only limited on-farm studies on the health performance of commercial dairy breeds. The aim of this longitudinal study was to quantify the incidence and identify predictors of calf morbidity and mortality from birth to 6 months of age in urban and peri-urban dairy farms of Northwest Ethiopia. A total of 439 calves aged below 6 months from 174 dairy farms were included in the study. We collected data on 35 potential risk factors to determine their effect on calf morbidity and mortality in the area. Kaplan–Meier survival analysis was used to summarize survival probability. The Cox proportional hazard regression model with shared frailty to account for unmeasured herd-specific heterogeneity was also used to identify and quantify factors associated with time to morbidity and mortality. Among 439 calves enrolled for 6 months of follow-up period, a total of 141 morbidities and 54 mortality events were recorded. This gives an overall morbidity and mortality incidence rates of 64 per 100-calf 6-months at risk (risk rate of 47.3%) and 19 per 100-calf 6-months at risk (risk rate of 17.9%), respectively. Diarrhea was the most frequent calf health problem with a risk rate of 25.2%. It was the cause of death for 33.3% of all the 54 calf deaths. Next to diarrhea, pneumonia ranked second with risk rate of 8.6% and was responsible for death of 12.9% of all the 54 calf deaths. Among 35 potential risk factors, calf age, vigor status at birth, calf breed, colostrum ingestion, and herd size were significant (*p* < 0.05) predictors of calf morbidity and mortality. The Cox-shared frailty model revealed that the herd frailty component had no significant effect on hazard estimates of the covariates of all-cause morbidity and mortality. This implies that the dairy herds participated in the study were homogeneous in the distribution of unmeasured random effects. In conclusion, the magnitude of calf morbidity and mortality was higher and above economically tolerable level in this study. This could impede the success of Ethiopia's dairy development initiative in general, and the livelihood of smallholder dairy producers in particular. Therefore, educating farmers aimed at mitigating the identified risk factors can reduce calf morbidity and mortality in the study areas.

## Introduction

Ethiopia has formulated policies and strategies to boost livestock production and productivity to meet the high demands for animal source foods (ASF) driven by rapid human population growth, increase in per capita income, and accelerated urbanizations. The country has launched key livestock development initiatives; the growth and transformation plan (GTPII-2015–2020), livestock master plan (LMP: 2015–2021), and the 10-years strategic plan for agriculture (2021–2030) to accelerate the country's livestock development in general and transform the dairy sector in particular (1). The LMP highlighted that, if no intervention is made to the dairy sector productivity, there will be a 24% (1,987 ML) deficit of cow milk by 2028 (2).

These policies are the basis for the ongoing dairy development initiatives and the booming of commercial and smallholder market-oriented urban and peri-urban dairy farms in the country (1, 3). These farms which keep both crossbred and exotic dairy breeds of cows have been expanding to peri-urban and urban areas in response to the acute shortage of dairy products (3). Nevertheless, the success of such initiatives depends, among other things, on the raising of sufficient healthier heifers for the replacement of improved dairy herds. Few studies from central and southern Ethiopia reported a high prevalence of calf health problems, which substantially affected the rising of sufficient dairy heifers (4–8). Urban and peri-urban dairies which keep high grade cows in Ethiopia, are usually associated with reproductive inefficiency, poor calf survival rate, increased susceptibility to disease, such as mastitis, lameness, pneumonia, and ketosis (3, 9).

Globally, one of the most important indicators of the health status in dairy farms is the frequency of morbidity and death, especially of calves during their first 6 months of life (10). The high incidence of calf morbidity and mortality incurs great economic loss to dairy producers associated with death loss, treatment cost, decreased lifetime productivity, and limited dairy herd expansion and genetic selection (11). The derivers of calf morbidity and mortality are multifactorial and involve a complex interaction of the management practices and environment, infectious agents, and the calf itself (12). Calf health and performance improvements can be achieved through the development and application of sound dairy calf health and management practices. These include proper colostrum management, quality nutrition, good housing, sanitation of the calf's environment and feeding utensils, and control of potential disease carriers (13).

However, knowledge of incidence and risk factors of calf morbidity and mortality are required for developing and applying such intervention measures (14, 15). Although urban- and peri-urban market-oriented commercial dairy farms have been emerging in northwestern Ethiopia, there are limited epidemiological reports on calf morbidity and mortality in urban and peri-urban dairy farms of Bahir Dar milk-shed. Therefore, the objectives of this longitudinal study were to (i) estimate the incidence of calf morbidity and mortality, and (ii) identify and quantify causes and predictors of calf morbidity and mortality from birth to six-months of age in urban and peri-urban dairy farms of Bahir Dar milk-shed, northwestern Ethiopia.

## Materials and Methods

### Study Area Description

**Figure 1:**
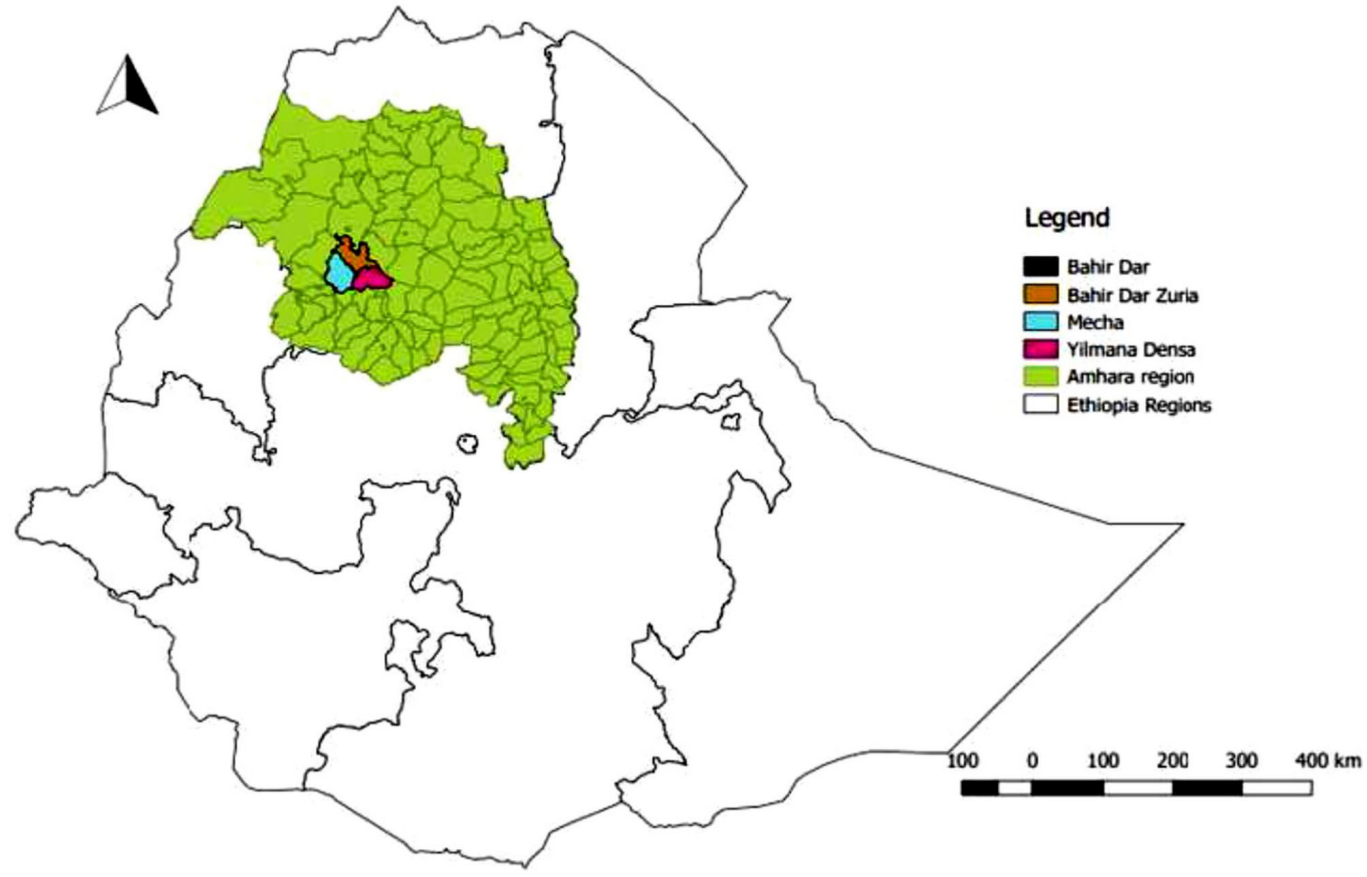
Location map of the study area.

This study was conducted in urban and peri-urban dairy farms found in Bahir Dar milk-shed of Amhara regional state, northwest Ethiopia (Figure 1). The study comprises three districts (Bahir Dar Zuria, Mecha, and Yilmana Densa) from West Gojjam Zone and Bahir Dar city from Bahir Dar special zone. Bahir Dar milk-shed is one of the eight major milk-sheds in Ethiopia, where several market-oriented smallholder and medium-to-large sized commercial dairy farms are emerging (16). In these areas, about half a million smallholders are engaged in dairy production with estimated annual milk production of 46,710,335 L. The predominant production system in the milk-shed is mixed crop-livestock farming where cattle are the most important livestock species. The study area comprises about 1.4 M cattle (17).

Bahir Dar is the capital city of Amhara National Regional State, which is located 565 km northwest of Addis Ababa along the Upper Blue Nile River basin. Bahir Dar is situated at an altitude of 1,840 m above sea level (masl) and at a latitude of 11°36′ N and longitude of 37°23′ E. The other study districts are found within 40-km radius from Bahir Dar city. The calf sampling sites had an elevation range of 1,642–2,360 masl with a mean temperature value of 18.6°C in the range of 11.54–32.3°C and mean humidity of 53% in the range of 35.2–74.5% (18).

### Study Farms and Animals

The study animals were dairy caves <6 months of age kept in the urban and peri-urban dairy production system in Bahir Dar milk-shed. Dairy farms were categorized as (i) urban and peri-urban [based on location, spatial land use, and integration with crop production as described by Tegegne and Gebrewold (3)] and (ii) smallholder [having 1–20 cattle(s) of all ages and sexes], and (iii) large farms [≥20 cattle of all ages and sexes based on herd sizes as described by Muraguri et al. (19)].

### Study Design and Sampling Strategy

A cross-sectional herd-level questionnaire survey followed by a calf-level prospective cohort study design was conducted on calf morbidity and mortality. A purposive sampling was used to select the study areas based on the availability of lactating dairy cows. Accordingly, three districts (Bahir Dar Zuria, Mecha, and Yilmana Densa) from West Gojam Zone and Bahir Dar city from Bahir Dar special zone were selected.

Member list of four dairy cooperatives found in Bahir Dar city and Bahir Dar Zuria district was used as a sampling frame to select farms. In both Bahir Dar city and Bahir Dar Zuria districts, 166 smallholder and commercial dairy farms organized under four dairy cooperatives were available. Whereas at Mecha and Yilmana Densa districts, the smallholder farms were registered at district agricultural office. The member list of the dairy cooperatives and district dairy farm records was used as sampling frame for the study. Simple random sampling technique using random numbers generated by Microsoft Excel RAND function was employed to select dairy farms from the sampling frame.

Accordingly, a total of 174 dairy farms (88 from Bahir Dar City and Bahir Dar Zuria, 51 from Mecha, and 35 from Yilmana Densa districts) were selected and enrolled for the study. When a selected member farmer did not have calf or pregnant cows with due calving date in the 6-months cohort period, it was then replaced by another dairy farmer mostly from the nearby area.

### Sample Size Estimation

Cluster sampling was used to select calves for the study. Dairy farms selected above were considered as clusters (primary sampling units) and calves were secondary sampling units. The sample size was calculated using a simple random sampling formula as provided in (20). Using expected morbidity/mortality of 50% (as there was no previous report in the present study area), 5% required precision and 95% confidence levels, the minimum required sample size was 384. However, according to Bennett et al. (21), the sample size estimated for simple random sampling was adjusted for the cluster sampling by multiplying it with the design effect.


$$\begin{array}{l} {\text{D}}_{\text{eff}}=1+\left(b-1\right)\;\times \;\rho \end{array}$$

where *D*_eff_ is the design effect, *b* is average cluster size, and ρ is *rho* (intra-cluster correlation). Assuming a value of intra-cluster correlation (ρ) of 0.2 (21), the average cluster/calf herd size of 1.72, the design effect was 1.14. Accordingly, the total sample size was 439; and the calves were proportionally drawn from each study district.

### Calf Recruitment and Follow-Up

A total of 439 (298 zebu x Holstein–Frisian crossbred and 141 local Zebu) calves from 174 dairy farms were enrolled and followed up for 6 months. The calves <1 month of age at the initial visit and whose disease history and date of birth known were recruited retrospectively and included into the prospective cohort. Other calves were recruited progressively as they were born within the selected farms during the study period. The recruited calves and those born after the initial visit were ear-tagged. All selected calves were regularly visited on weekly basis by the investigator as well as by assigned enumerator until the calves reached 6 months of age. In the interim period, calf health problems were monitored by the trained veterinarian and calf attendant. When problems are suspected, the researcher was called on for further examination and identification of the causes. When the calf loss happened during the follow-up period, the date and the reason of loss was recorded.

### Data Collection

A questionnaire was administered to 174 dairy farmers to collect herd-level data pertaining to farm characteristics and calf management practices, such as calf colostrum management, calf housing, healthcare, and overall feeding management practices. Whereas, calf-level factors were collected longitudinally during the follow-up period. These included recording of genealogy of the calf, place of birth, calving events, colostrum administration, initial housing, routine management practices applied to the calf, and calf health problems (morbidity and mortality incidents) that were observed during the follow-up period. Morbidity was defined as any recognizable clinical signs which warranted therapeutic intervention during the course of follow-up period, and mortality was defined as any observed death after 24 h of live birth.

The causes of illness were determined based on clinical examination by the trained veterinarian assigned for the follow-up. Some cases were diagnosed as a specific disease, such as lumpy skin disease and rabies, and others at only syndrome level like diarrhea and pneumonia.

According to dairy producers' response, the newborn calves were grouped into two categories based on whether they were freely allowed to suckle colostrum or not at birth. This included (i) colostrum-fed, when a calf freely allowed to suckle the colostrum at birth and (ii) colostrum-unfed when a calf was not allowed to suckle colostrum (when the calf was allowed to suckle the dam only after the first colostrum is milked and discarded). Likewise, the newborn calves were also grouped into two based on their vigor status at birth. Good vigor calves, which stood or attempted to stand and suckle the dam within 1 h after birth and poor vigor calves, which stayed recumbent and did not attempt to suckle the dam within 1 h after birth (22).

## Data Analysis and Statistical Modeling

### Estimation of Morbidity and Mortality Rates

The data were checked, coded, and entered into Microsoft Excel 2016 spreadsheet and exported to STATA version 15 (Stata Corp LLC. Texas USA, 2017) for data analysis. As calves were recruited at different times and were followed for different periods of time, incident rate (true rate) was used in describing diseases occurrences (19). The incidence rates (IRs) were calculated as follows:


$$\begin{array}{l} \text{Incidence rate }(\text{IR})=\text{number of events occurred during}\\ \text{ observation period/total calf days at risk} \end{array}$$

The numerator is the number of occurrences of the outcome of interest during the follow-up period and the denominator is the number of calf-days at risk during the same period. When a calf is recovered from an illness or the clinical sign of the disease is disappeared, it was considered to be at risk for another illness. Two or more renewed or relapsed cases of the same disease condition were considered as different cases in calculating the incidence of that disease condition as far as the second case occurred after the disappearance of the clinical sign of the first. However, the prevalence was used to estimate the frequency of congenital defects in calves, as these defects were time independent for individual calves and recorded only in the first visit of individual calves. To facilitate result comparisons with other similar previous studies which are usually reported in percentages (risk rates), the IRs calculated for mortality, morbidity and other relative morbidities were converted to risk rates based on the formula RR = 1 – *e*^−True Rate^ (23).

### Survival Analysis

The duration until the occurrence of morbidity and mortality events was monitored until 180 days of age to construct an actuarial life table and Kaplan–Meier (K–M) survivorship curves. We used Cox proportional hazard regression model (referred as Cox regression hereafter) with shared frailty to account for unmeasured herd level effects to identify and quantify factors associated with time to occurrence of morbidity and mortality. The Cox regression model (24) provides that the hazard for the *j*th subject in the data is


$$\begin{array}{l} h\left(t\right)=ho\left(t\right)\exp\left(\beta 1x1+\beta 2x2+...\beta pxp\right) \end{array}$$

where *t* represents the survival time and *h*(*t*) is the hazard function determined by a set of *P* covariates (*x*_1_, *x*_2_, …., *x*_p_), β_1_, β_2_, …, β_p_ are the regression coefficients to be estimated from the data and measure the effect size of covariates. The *ho*(t) is the baseline hazard and represents the hazard when all of the predictors (covariates) *x*_1_, *x*_2_, and *x*_p_, are equal to zero.

The Cox regression model implicitly assumes that the hazards are proportional, in which relative hazards remain constant over time with different covariate levels. However, it is important to consider the population as heterogeneous since it is impossible to include all relevant risk factors. Such unobserved heterogeneity and dependence of survival times in clustered data, can be accounted through inclusion of random effects (frailties) in the model (25, 26). Frailty is a latent random effect that enters multiplicatively on the hazard function (27). As the study subjects (calves) in this study were taken from different (174) dairy farms/clusters, there may be unobserved heterogeneity and survival data may be correlated at herd level. Hence, herds/clusters were included in the Cox regression model as random effects to account herd specific unobserved heterogeneity.

For a Cox regression shared frailty model, the data were organized according to Cleves et al. and Fagbamigbe et al. (28, 29), as *I* = 1, …, *n* groups (clusters) with *j* = 1, …, *k* subjects (calves) within group *i*. Assume conditional on frailty *V*_i_, for the *j*th subject in the *i*th group, the hazard is


$$\begin{array}{l} h_{ij}\left(t\right)=ho\left(t\right)\alpha i\;\exp\left(\beta 1x1+\beta 2x2+\ldots \beta pxp\right) \end{array}$$

where α_i_ is the group (herd) level frailty. The frailties are unobservable positive quantities and are assumed to have mean 1 and variance θ, to be estimated from the data (28, 30). For *Vi* = logα_*i*_, the hazard can also be expressed as


$$\begin{array}{l} hij\left(t\right)=ho\left(t\right)\exp\left(\beta 1x1+\beta 2X2+\ldots \beta pxp+Vi\right) \end{array}$$

The log frailties, *Vi*, are analogous to random effects in standard linear models. Here, *V*_i_ '*s* reflect variability, and this shows heterogeneity of risks between herds. From the model, the value of 0 frailty reflects those calves from the same herds are independent (there is no frailty). Therefore, large variance values (θ > 0) indicate high heterogeneity between herds and a greater correlation between calves within the same cluster/herds.

#### Test of Proportional Hazards Assumptions and Model Building Strategy

Assumptions for K–M survival curves and proportional hazards were tested according to Kuitunen et al. (26) and Cleves et al. (28), using K–M graphical methods and the Schoenfeld test. The hypothesized predictor variables were screened biologically and statistically until the minimum number of events per variable (EPV), i.e., 0.1 EPV for the multivariable Cox regression model was achieved (31, 32). First, the predictor variables were screened for their biological plausibility and relevance, and then those variables that passed the first biological relevance evaluation were subjected to statistical screening using univariable Cox regression at *p* < 0.25 (33, 34). Because the number of variables that passed these two screenings (biological and statistical) were more than the EPV requirement, they were further evaluated for their more biological relevance and statistical significance according to the recommendation of Chowdhury et al. (33) and screened until the required minimum EPV was achieved. Finally, the variables that passed all screenings were included for the multivariable Cox regression model. Those screened variables in multivariable Cox-regression were evaluated for their independent effect at *p* < 0.05 after the effects of the other variables were controlled. The final fitted model was achieved through stepwise backward elimination of insignificant variables (*p* ≥ 0.05) for each outcome variable.

## Results

### Description of Dairy Farms Based on Cross-Sectional Questionnaire Survey

Among total dairy producers interviewed in Bahir Dar milk-shed, 60.9% (106/174) and 39.1% (68/174) of them were from urban and peri-urban dairy farms, respectively. Dairy production served as a primary source of livelihood for 39.7% (69/174) of smallholder farmers. Average age of respondents was 44.3 (SD ± 11.2) years, in which 90.2% (157/174) and 9.8% (17/174) of them were male and female respondents, respectively. The respondents, on average, owned 1.7 (SD ± 1.6) pre-weaned calves. The local and crossbred cows (local crossed with HF: >50%) yielded an average of 1.8 (SD ± 0.6) and 10.6 (SD ± 4.1) L/day in the peak lactation period, respectively (Table 1).

**Table 1:** Dairy farm characteristics in urban and peri-urban dairy farms of Bahir Dar milk-shed, Northwest Ethiopia.

**Farm attributes**	***N***	**Minimum**	**Maximum**	**Mean ±SD**
Age of house hold/respondent (year)	174	20	77	44.3 ±11.2
Number of lactating cows	174	1	14	2.3 ± 2.4
Total cattle holding	174	2	43	8.1 ± 6.62
Number of pre-weaned local calves (<12 m)	174	0	2	0.4 ± 0.6
Number of pre-weaned crossbred calves (<6 m)	174	0	14	1.4 ± 1.6
Total pre-weaning calf herd size (local and cross)	174	1	14	1.7 ± 1.6
Birth weight for local calves (kg)	80^a^	16	29	22.8 ± 2.7
Birth weight for crossbred calves (kg)	57^b^	21	41	29.1 ± 4.6
Milk yield in peak lactation period for local cows (L/day)	174	1	4	1.8 ± 0.6
Milk yield in peak lactation period for crossbred cows (>50% EBL: L/day)	174	4	24	10.6 ± 4.1

*N, number of respondents; a, number of local calves; b, number of crossbred calves; EBL, Exotic blood level*.

Calf morbidity and mortality were ranked by 28.7% (50/174) of producers as a primary dairy health problem. Of which, 17.2% (30/174) and 11.5% (20/174) of respondents from urban and peri-urban dairy, respectively, have experienced calf mortality during the last 1 year. More than two-third (68.4%, 119/174) of dairy owners had awareness about the advantage of colostrum feeding to newborn calves. The remaining 31.6% (55/174) of respondents believed that colostrum causes gastric disorder and retention of fetal membrane, and hence they often discard the colostrum and prevent their calves to suckle colostrum. Among the colostrum providers, 96% (167/174) of them use the suckling method and the rest 4.6% (8/174) use hand feeding. About 80% (139/174) of the farmers separate calves from their dams after1-3 days whilst 19.5% (34/174) of the owners separate immediately after first nursing. Separate calf pen was provided by 57.5% (100/174) of producers. Rest of the producers kept their calves in the cowshed. Once the calf was separated from the dam, 96% (167/174) of dairy farms fed residual milk twice a day.

No special starter feed was used in any of the farms; rather the same feed given to cows was given to calves. These include straw, crop residue {mainly maize, millet, and teff [*Eragrostis tef* ] and concentrate mixture [wheat bran and Nug (*Guizoitia abyssinica*) cake]}. Other non-conventional feeds, such as local beer by-products (*atela* and *brint*), were also often provided as alternative feed resources. Stall-feeding was practiced by 64.4% (112/174) of the producers and 35.6% (62/174) practice grazing with a concentrate mixture supplement for their calves. Age to introduce non-milk feed (weaning age) varied from farm to farm.

In most urban dairy farms, the average weaning age was 6.8 months in crossbred calves, while a relatively extended weaning age of 8 months was recorded in peri-urban dairy farms. The average weaning age for local calves was 12 months.

### Longitudinal Prospective Cohort Study

#### Cohort Dynamics During the 180 Days of Follow-Up

**Figure 2:**
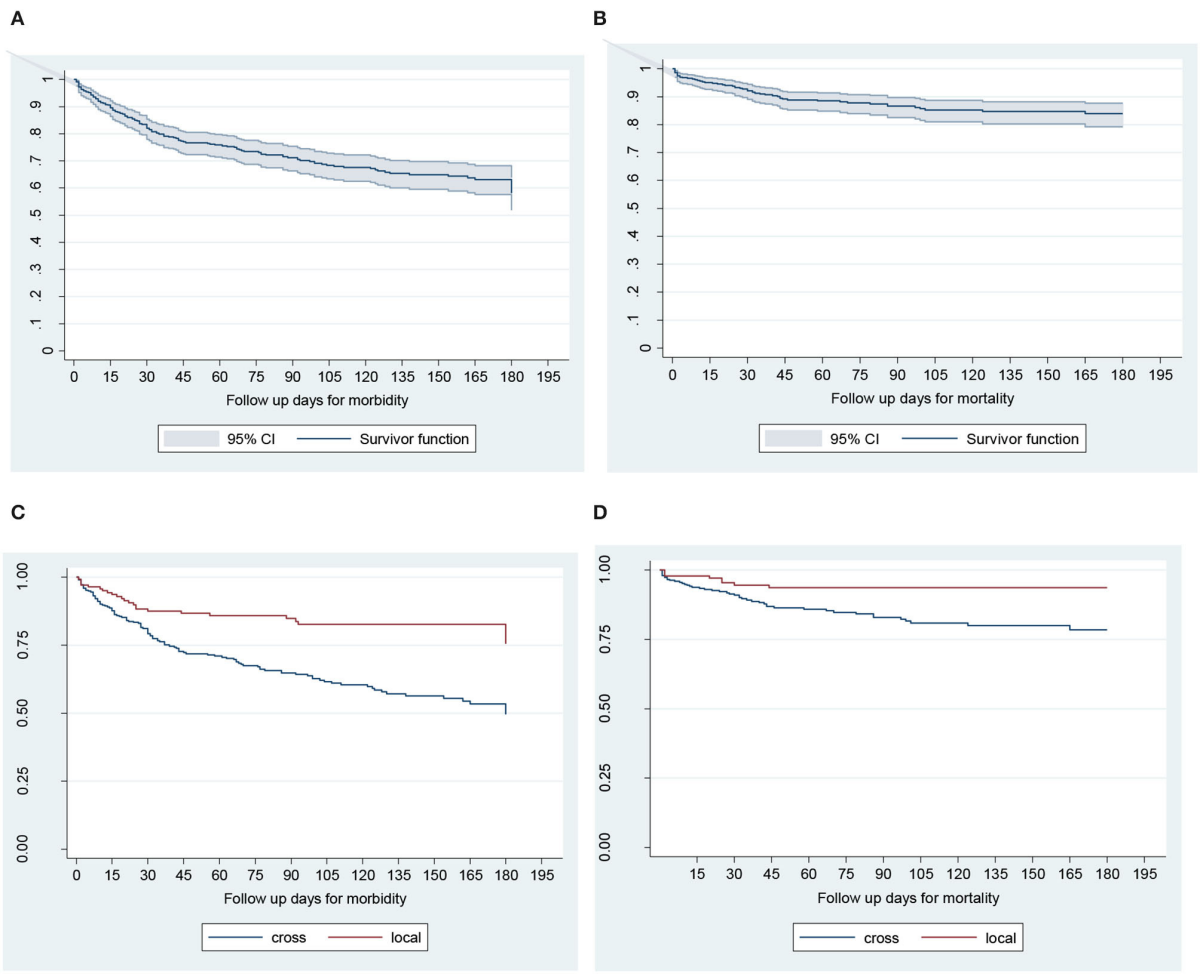
Kaplan–Meier survival curves for time to morbidity **(A)**, mortality **(B)**, effects of breed on time to morbidity **(C)**, and mortality **(D)** in calves during the first 180 days of life in Bahir Dar milk-shed, Northwest Ethiopia.

A total of 439 dairy calves were followed for 180 days. All calves contributed a total of 49, 237-calf days at risk, which is also equivalent to 274-calf 6-months at risk. Slightly more females 233 (53%) were borne to join the follow-up than males 207(47%) during the 180 days follow-up period. Overall, 54 calves were died and 7 were sold before the study cohort ends. The total exit rate was 13.9% (61/439), of which 32 (7.3%), and 29 (6.6%) were females and males, respectively. The overall cohort dynamics and survival pattern during 180 days study period are shown in Table 2; Figure 2.

**Table 2:** Actuarial life table summary of 439 calves enrolled for calf morbidity and mortality study in Bahir Dar milk-shed, Northwest Ethiopia.

**Interval (days)**	**Number falls the interval**	**Censored**	**Cases**	**Survival probability**	**Stand. Error**	**95% Confidence interval**
**Morbidity**						
0-30	439	41	70	0.8327	0.0182	0.7934-0.8652
30-60	328	39	29	0.7545	0.0216	0.7091-0.7938
60-90	260	36	15	0.7077	0.0234	0.6591-0.7507
90-120	209	37	11	0.6704	0.0249	0.6190-0.7166
120-150	161	35	6	0.6424	0.0264	0.5881-0.6915
150-180	120	29	3	0.6241	0.0277	0.5674-0.6757
180-	88	81	7	0.5322	0.0398	0.4511-0.6066
Total	-	298	141	-	-	-
**Mortality**
0-30	439	81	30	0.9247	0.0132	0.894-0.9468
30-60	328	54	14	0.8817	0.0169	0.8440-0.9108
60-90	260	46	5	0.8631	0.0185	0.8223-0.8952
90-120	209	45	3	0.8492	0.0198	0.8055-0.8838
120-150	161	40	1	0.8432	0.0206	0.7979-0.8791
150-180	120	31	1	0.8351	0.0219	0.7869-0.8733
180-	88	88	0	0.8351	0.0219	0.7869-0.8733
Total	-	385	54	-	-	-

#### Incidence Rate of Morbidity and Mortality

The overall morbidity and mortality IRs were 64 per 100-calf 6 months at risk (risk rate of 47.3%) and 19 per 100-calf 6-months at risk (risk rate of 17.9%), respectively. The leading calf health problem in the study area was calf diarrhea with IR of 29 per 100-calf 6-months at risk (risk rate of 25.2%), followed by pneumonia with IR of 9 per 100 calf 6-months at risk (risk rate of 8.6%). The incidences of other diseases of the calves were relatively low (Table 3).

**Table 3:** Summary of calf-time at risk and IR of major calf health problems in urban and peri-urban dairy farms of Bahr Dar milk-shed, Northwest Ethiopia (*N* = 439).

**Major calf health problems**	**Duration of study (days)**	**Calf days at risk**	**Number of new cases**	**IR/Calf 6-months at risk**	**Risk rate (%)^**a**^**
Morbidity, all cause	180	39,844	141	0.64	47.3
Mortality, all cause	180	49,237	54	0.19	17.9
**Relative morbidities**
Diarrhea	180	43,942	71	0.29	25.2
Pneumonia	180	47,615	23	0.09	8.6
Omphalitis (Naval ill)	180	47,459	14	0.05	4.9
Septicemia	180	48,917	6	0.02	1.9
Lumpy Skin Disease	180	48,725	10	0.04	3.9
Rabies	180	49,172	2	0.01	0.9
Miscellaneous	180	46,871	15	0.05	4.9
Congenital defects	-	439^b^	5		1.1^c^

*b, number of calves; c, prevalence; a, Risk rates estimated from true rates using the formula*,

*Risk rate = 1 – e^−True Rate^ (23)*.

#### Survival Probability of Time to Morbidity and Mortality

The cumulative survival proportion of calves for all-cause morbidity and mortality (which are the converses of cumulative morbidity and mortality) were estimated using K–M survival curve (Figure 2). The cumulative calf morbidity at 7, 30, 90, and 180 days was 6, 18, 29, and 47%, respectively (Figure 2A). Similarly, the cumulative calf mortality for the corresponding periods was 93, 5, 11, and 16%, respectively (Figure 2B). Overall, the 6 months cumulative morbidity and mortality proportions were 47 and 16%, respectively (Figures 2A,B; Table 2). During the 180 days of monitoring, the survival probability for both morbidity and mortality was higher for local breed than crossbreed calves (Figures 2C,D).

#### Modeling of Predictors for Calf Morbidity and Mortality

##### Predictors of All-Cause Morbidity

From 35 potential predictor variables for morbidity considered in the study (Annexure IV), 17 of them were biologically evaluated and passed for statistical screening using univariable Cox regression at *p* < 0.25 to be included in the multivariable Cox regression model. The predictors that were significantly (*p* < 0.25) associated with all-cause morbidity based on univariable Cox regression are shown in Table 4. Of those significant predictors included in the univariable analysis, three of them, namely, birth time, type of agriculture, and calf caretaker, were dropped based on their biological relevance and statistical significance to achieve the minimum EPV for the multivariable Cox regression and the remaining 14 predictor variables were analyzed by multivariable Cox regression with shared frailty (Table 4). However, only calf age, vigor status at birth, calf breed, colostrum feeding, and study location were found significantly associated with all-cause morbidity (Table 5). The hazard estimates and associated statistics for covariates and the variance of frailty (clusters/herds) are shown in Table 5.

**Table 4:** Predictor variables significantly associated with all-cause morbidity based on univariable Cox regression screening analysis in Bahir Dar milk-shed, Northwest Ethiopia (*N* = 439).

**Variables**	**Categories**	HR^*^	**95% CI for HR**	***P***
Calf breed	Cross vs. local	2.51	1.64-3.85	<0.001
Calf age	Young(<3 m) vs. old (≥3 m)	2.19	1.50-3.20	<0.001
Calf vigor status at birth	Good vs. poor	0.14	0.09-0.24	<0.001
Colostrum feeding	Unfed vs. fed	1.52	1.02-2.28	0.041
Ease of birth	Dystocia vs. normal	1.50	0.97-2.32	0.063
Calf barn hygiene	Clean vs. unclean	0.77	0.54-1.12	0.123
Birth time	Day vs. night	0.12	0.11-0.42	0.145
Dam parity	Multiparous vs. primiparous	0.71	0.47-1.11	0.136
Herd size	Small (<20) vs. large (≥20)	0.79	0.55-1.12	0.082
Type of agriculture	Mixed vs. livestock	1.25	0.88-1.77	0.213
Calf accommodation	Separate vs. group	0.61	0.43-0.861	0.005
Method of colostrum feeding	Bucket vs. suckling	1.27	1.02-1.59	0.030
Dam vaccination history	Unvaccinated vs. vaccinated	1.25	1.05-1.51	0.081
Dairy as primary source of income	Yes vs. no	0.48	0.34-0.69	0.031
Dairy production System	Urban vs. peri-urban	2.20	1.57-3.07	<0.001
Calf care taker	Hired vs. owner	1.25	0.89-1.75	0.187
Study location/Districts	Yilmana Densa	Ref	-	-
	Bahir Dar City	3.0	1.39-6.61	0.005
	Bahir Dar Zuria	1.5	0.61-3.00	0.460
	Mecha	1.4	0.63-3.51	0.364

* *Hazard ratio*.

**Table 5:** Predictor variables significantly associated with the risk of all cause-morbidity in the Multivariable Cox regression analysis in Bahir Dar milk-shed, Northwest Ethiopia (*N* = 439).

**Variables**	**Categories**	HR^*^	**95% CI for HR**	***p***
Calf age	<3 months vs. ≥3months	2.23	1.51-3.29	<0.001
Calf breed	Cross vs. Local	1.84	1.15-2.95	0.011
Calf vigor status at birth	Poor vs. Good	4.45	2.66-7.46	<0.001
Colostrum feeding	Unfed vs. Fed	1.86	1.19-2.97	0.007
Study location	Yilmana Densa	Ref.	-	-
	Mecha	1.85	0.77-4.43	0.168
	Bahir Dar Zuria	1.61	0.711-3.64	0.253
	Bahir Dar City	2.79	1.24-6.27	0.013
Theta(θ)^a^	-	1.13e-07	-	0.49

* *Haza ratio*,

a *Variance of the unobserved frailty parameter: LR test of theta = 0 chibar2(01) = 1.5e^−05^ Prob > = chibar2 = 0.49*.

The hazard for morbidity was higher in younger (<3 months) (HR = 2.23; 95% CI; 1.51–3.29; *p* < 0.001), crossbred (HR = 1.84: 95% CI; 1.15–2.95; *p* = 0.011) and poor vigored (HR = 4.45; 95% CI; 2.66–7.46; *p* < 0.001) calves. After adjustment, the hazard for morbidity was 1.86 times higher (95% CI; 1.19–2.97; *p* = 0.007) in those calves who did not ingest their first colostrum at birth. As indicated from Table 6, inclusion of herd-specific frailty term (θ = 1.13*e*^−07^; *p* = 0.49) in the model had no significant (*p* > 0.05) effect on the magnitude of the covariates estimated for all-cause morbidity.

**Table 6:** Predictor variables significantly associated with the incidence of all-cause mortality based on univariable Cox regression screening analysis in Bahir Dar milk-shed, Northwest Ethiopia (*N* = 439).

**Variables**	**Categories**	HR^*^	**95% CI for HR**	***p***
Calf age	≥3 months vs. <3 months	0.03	0.01-0.08	<0.001
Calf breed	Local vs. cross	0.32	0.15-0.68	0.003
Calf vigor status at birth	Good vs. poor	0.06	0.04-0.12	<0.001
Colostrum feeding	Fed vs. unfed	0.46	0.25-0.83	0.011
Dam parity	Multiparous vs. primiparous	0.87	0.79-0.96	0.065
Ease of birth	Dystocia vs. normal	2.11	1.13-3.93	0.039
Calf care taker	Hired vs. owner	1.43	1.08-1.91	0.096
Herd size	Large (≥20) vs. small (<20)	1.82	1.06-3.08	0.011
Dairy production system	Per-urban vs. urban	0.51	0.29-0.86	0.051
Type of Agriculture	Mixed vs. livestock	0.41	0.21-0.79	0.081
Study location	Yilmana Densa	Ref.	-	-
	Mecha	1.01	0.091-11.15	0.992
	Bahir Dar Zuria	5.12	0.69-37.97	0.110
	Bahir Dar City	8.00	1.08-58.87	0.043

* *Hazard ratio*.

##### Predictors of All-Cause Mortality

As for morbidity, from 35 potential predictor variables of mortality considered in the study (Annexure IV), 11 of them were biologically evaluated and passed for statistical screening using univariable Cox regression at *p* < 0.25 to be included in the multivariable Cox regression model. Predictors that were significantly (*p* < 0.25) associated with all-cause mortality based on univariable Cox regression are shown in Table 6. Of those significant predictors included in the univariable analysis, five of them, namely, calf age, calf breed, calf vigor status at birth, colostrum feeding, and herd size were selected based on their biological relevance and statistical significance to achieve the minimum EPV for the multivariable Cox regression (Table 7). Accordingly, calf age, calf vigor status at birth, calf breed, colostrum feeding, and herd size were found to be significant predictors of all-cause calf mortality (Table 7). The hazard for mortality was lower in older (≥3 months) (HR = 0.02; 95% CI; 0.01–0.06; *p* < 0.001), good vigored (HR = 0.11; 95%CI; 0.05–0.25; *p* < 0.001) and local breed (HR = 0.17; 95% CI; 0.07–0.43; *p* = 0.001) calves than that of younger (<3 months), poor vigored, and crossbred counterparts, respectively.

**Table 7:** Predictor variables significantly associated with risk of all-cause mortality in the multivariable Cox regression analysis in Bahir Dar Milk-shed, Northwest Ethiopia.

**Variables**	**Categories**	HR^*^	**95% CI for HR**	***p***
Calf age	≥3 months vs. <3 months	0.02	0.01-0.06	<0.001
Calf breed	Local vs. Cross	0.17	0.07-0.43	0.001
Calf vigor status at birth	Good vs. Poor	0.11	0.05-0.25	<0.001
Colostrum feeding	Unfed vs. Fed	5.28	2.25-12.41	<0.001
Herd size	Large (≥20) vs. Small (<20)	2.32	1.22-4.41	0.003
Theta (θ)^a^	-	0.573	-	0.083

* *Hazard ratio*,

a *Variance of the unobserved frailty parameter: LR test of theta = 0 chibar2(01) = 1.92 Prob > =chibar2 = 0.083*.

After adjusting the effect of other variables constant, the hazard for mortality was 5.28 times higher (95% CI; 2.25–12.41; *p* < 0.001) in those calves who were not allowed to suckle colostrum at birth. Likewise, the hazard for mortality was 2.32 times higher (95% CI;1.22–4.41; *p* = 0.010) in calves kept in large-sized dairy herds when compared to smallholder dairy farms. The hazard estimates and associated statistics for covariates and the variance of frailty (clusters/herds) are shown in Table 7. The variance of frailty (clusters/herds) (θ = 0.573; *P* = 0.083) revealed that the herd-specific frailty component had no a significant effect (*p* > 0.05) on the magnitude of the covariates estimated for all-cause mortality.

## Discussion

### Incidence of Morbidity and Mortality

This study showed high morbidity and mortality of calves in the first 6 months of calves' age in urban and peri-urban dairy farms of Bahir Dar milk-shed, North-western Ethiopia. The overall morbidity and mortality incidence risk rates were estimated to be 47.3 and 17.9%, respectively. The 47.3% morbidity finding in this study was within the range of calf morbidity of 29.3% (7) and 30.9% (8) in southern Ethiopia and 62% in central Ethiopia (6). The morbidity of a calf has an overarching effect on its future survival and productivity (10, 11) and hence the observed high morbidity can significantly impair diary productivity in the area.

The 17.9% risk of mortality recorded in this study was comparable with previous reports of 18.5% from Oromia and Amhara regions in Ethiopia (35) and in different parts of Africa (19, 36). However, the present mortality risk was found relatively lower than the previous Ethiopian reports of 20% in and around Addis Ababa (4), 22% mortality in Ada'a Liben district of Oromia (6), 25% mortality reported from Adami Tulu (37), 30.7% from Gozamen and Bahir Dar Zuria districts (38) and 53% from Ada Berga state farm (39). However, it was found higher than the incidence risk of calf mortality reports of 9.3% in Hawassa (7) and 8.6% in southern Ethiopia (8). Calf mortality has been reduced in the western modern dairy production systems, such as 5% in Norway (40), 3% in Sweden (41), and 2–6% in Britain (10). Some researchers have reported that the recommended economically tolerable level for calf mortality is 3–5% (42); hence, Ethiopia has a long way to go to reduce its calf mortality level.

The discrepancy between the present and previous reports in Ethiopia and elsewhere might be attributed to variations in the length of follow-up period with respect to calf age, calf and herd-level risk factors, herd size, management practices, breed of the calf, and agro-climatic conditions. For instance, most of the previous studies in Ethiopia have used a less powerful study design, i.e., a cross-sectional study (37, 38) and estimated calf morbidity and mortality using prevalence which did not properly show the risk or force of morbidity. Whereas other reports were based on studies in research stations, commercial dairy farms, and government ranches with relatively large herd sizes and high crossbred and exotic dairy animals which could be associated with increased risk of mortality occurrence (37, 39). Moreover, the lower calf mortality rate from western dairy production might be associated with improved dairy farm management practices. We believe that our study is reliable, as we used a more powerful longitudinal study as per Caruana et al. (43) and screened several risk factors under smallholder farmers setting.

Among cause-specific morbidities recorded, calf diarrhea (25.2%) and pneumonia (8.6%) were the first and second calf health problems and the main causes of mortality in the study areas. This finding is consistent with reports from Ethiopia (5, 6, 8, 35) and abroad (40, 44, 45). Lack of separate calf pen and lack of adequate colostrum could be the major predisposing factors affecting the occurrence of calf diarrhea in the present study area. In this regard, 42.5 and 31.6% of dairy producers in the study area did not have separate calf pen, and adequate and timely colostrum-feeding practices, respectively, that might contribute to the observed high incidence of diarrhea. The association between calf diarrhea and calf-cow contact was reported by Trotz-Williams et al. (46), suggesting that leaving the calf with the dam for more than an hour after birth could increase the risk of diarrhea.

Pneumonia was the second most important disease of calves, which caused 12.9% of calf deaths. This finding was found in agreement with (45), who reported 13% of calf deaths associated with respiratory diseases in Iranian Holstein dairy herds. It was relatively lower than documented by Heinrichs (47) in which calf pneumonia accounts for 15% of calf mortality from birth to 6 months of age. A relatively low number of calves or animals per barn in this study might be the reason for relatively low incidence of pneumonia. Pneumonia may be a problem in crowded housing. We observed 1.1% prevalence of congenital defects (congenital blindness and absence of tail) in crossbred calves. It was lower than the previous report of 5% prevalence of congenital problems in dairy calves in Debre Zeit and its environs (48).

### Predictors of Calf Morbidity and Mortality

Calf age particularly below 3 months, calf vigor status at birth, calf breed, colostrum feeding, and herd size were the critical factors predicting all cause-morbidity and mortality. A high proportion of calf death (60–75%) occurs in the first week of life (6, 40, 41), which indicates the first few weeks of calf is critical age that needs most attention in calf rearing.

Calf breed was a significant risk factor, where crossbred calves could be at higher risk for morbidity and mortality. The higher morbidity and mortality in crossbred calves found in this study might be associated with the susceptibility of *Bos taurus* blood to climatic and disease stress in a tropical environment (49). We found that poor-vigored calves were at higher risk for morbidity and mortality than that of good vigored calves. The relationship between vigor status at birth and calf morbidity and mortality was previously documented (22, 47). Poor vigor at birth decreases the chance of sufficient colostrum intake and increases the risk of death (22). Inadequate colostrum ingestion was found significantly associated with calf mortality and morbidity. We found that calves that did not suckle colostrum at birth were at higher risk of mortality in this study. Many published studies have demonstrated the significance of adequate and optimal time of colostrum feeding *vis-a-vis* mortality and morbidity (50, 51). Colostrum feeding is the single most important management factor in determining calf health and survival (13). The hazard for mortality was higher in relatively large-sized dairy herds when compared to smaller herd sizes. This finding was supported by Santos et al. (52), who reported that herd size has a predictive value on calf mortality from birth to weaning.

The hazard for calf morbidity was significantly higher in Bahir Dar City when compared to Yilmana Densa district. A significant morbidity variation between the two districts could be attributed to differences in breed, herd size, and calf management practices. Most dairy farms in Bahir Dar city were relatively specialized ones, having higher herd sizes with high-grade dairy cows (>50% exotic blood level). Whereas dairy farms in Yilmana Densa district were smallholder ones having smaller herd sizes and local diseases resistance cattle breeds.

In general, the assessment of calf management of practice of farmers revealed that it was not up to the standard. About a third of respondents deny calves from taking colostrum on erroneous belief that it causes calf diarrhea. The remaining farmers also did not have knowledge on the role of colostrum feeding in calf's health. Almost all of the calves feed on residual milk. This makes difficult to estimate how much milk the calves are getting and whether it does meet their requirements. About half of the farmers housed their calves together with cows and this is likely to increase infection pressure on calves as reported by Trotz-Williams et al. (46). Therefore, training and extension on calf management should be considered to reduce calf morbidity and mortality in the study area.

## Conclusion

The study has revealed high morbidity and mortality rates of calves in urban and peri-urban dairy farms of Bahir Dar milk-shed, northwest Ethiopia. This morbidity and mortality magnitude is higher than economically tolerable level with a subsequent negative impact on the availability of healthy and productive replacement stock. This ultimately hinders the success of Ethiopia's dairy development initiative in general and income and livelihood of smallholder dairy producers of the study area in particular. Calf diarrhea and pneumonia were the predominant calf health problems responsible for the majority of calf illnesses and deaths. Evaluation of the effect of multitude of determinant factors (35 potential risk factors) on calf morbidity and mortality is performed in this study. Calf age, calf breed, calf vigor status at birth, colostrum feeding, and herd size are significant risk factors affecting calf morbidity and mortality in the study areas. Some of these risk factors, such as timing and volume of colostrum feeding, nevertheless, are amenable for intervention. Therefore, we recommend tailored interventions directed at improving management practices to reduce morbidity and mortality in the study area.

## Data Availability Statement

The original contributions presented in the study are included in the article/Supplementary Material, further inquiries can be directed to the corresponding author/s.

## Ethics Statement

The animal study was reviewed and approved by Ethics Committee of College of Veterinary Medicine and Agriculture, Addis Ababa University Reference Number VM/ERC/16/13/20. Written informed consent was obtained from the owners for the participation of their animals in this study.

## Author Contributions

YA, RA, WJ, and ZM conceived and designed the study and analyzed and interpreted the data. YA collected the data and drafted the manuscript. RA, WJ, and ZM revised the manuscript. All authors contributed to the article and approved the submitted version.

## Funding

This work was supported by the Department of Foreign Affairs, Trade and Development of Canada (DFATD), and Amhara Agricultural Research Institute (ARARI).

## Conflict of Interest

The authors declare that the research was conducted in the absence of any commercial or financial relationships that could be construed as a potential conflict of interest.

## Publisher's Note

All claims expressed in this article are solely those of the authors and do not necessarily represent those of their affiliated organizations, or those of the publisher, the editors and the reviewers. Any product that may be evaluated in this article, or claim that may be made by its manufacturer, is not guaranteed or endorsed by the publisher.
