# Supplementary material for: Incidence and Predictors of Calf Morbidity and Mortality From Birth to 6-Months of Age in Dairy Farms of Northwestern Ethiopia
Source: Front Vet Sci. 2022 May 23;9:859401. doi: 10.3389/fvets.2022.859401 (PMC9169041; doi:10.3389/fvets.2022.859401)
Supplement: Supplementary file 1 [file Table_1.DOCX]

**Supplementary Data**

# ANNEXES

Annex I. Questionnaire for herd level management data collection associated with dairy calf morbidity and mortality in Bahir Dar milk-shed.

*1. General information*

Date of interview………………………. Zone……………Woreda…….…….Kebele………..Village………….…..Tel…………

Altitude of the area (M.a.s.l) a) high altitude (>2000) b) mid altitude (1500-1800)

c) low altitude (<1500)

*2. Farm, household and land holding characteristics*

2.1. Name of the household head/respondent……………

2.2. Sex of house hold head a) male b) female

2.3. Age of house hold head………………

2.4. Marital status a) married b) single c)Widow d)Divorced

2.5. House hold educational status a) illiterate b) read and write c) elementary school

d) high school graduate e) professional,

If professional a) related to animal production b) unrelated to animal production

2.6. Family size: Male…….Female……….Total…………

2.7. System of Agricultural production a)livestock c)mixed crop- livestock

2.8. Land holding: Cultivable/farming land (ha)……Private grazing land (ha)…. Total……

*3. Dairy Production System*

3.1. What are your major livestock activities?

a) Dairy production b) Small ruminant production c) poultry production

3.2. Dairy production as a source of income a) primary b) secondary/side line activity

3.3. How long have you engaged in dairy production?...................

3.4. Dairy farm location

a) Urban b) peri-urban

3.5. Herd size and composition

| Herd composition | | local | Cross | Total |
| --- | --- | --- | --- | --- |
| Calves (<6m) | Male |  |  |  |
|  | Female |  |  |  |
| Calves (6-12m) | Male |  |  |  |
|  | Female |  |  |  |
| Heifers | |  |  |  |
| Lactating cows | |  |  |  |
| Dry cows | |  |  |  |
| Bull | |  |  |  |
| Bullock | |  |  |  |
| *Total herd size* | |  |  |  |

*4. Calf Management data*

4.1. Breeding methods used a) AI b) natural mating c) both

4.2. Calf caretaker (attendant)

4.2.1. Ownership a) owner (family member) b) hired

4.2.2. Sex a) male b) female

4.2.3. Experience a) <= 5 years b) >5 years

4.3. Pregnant cow management and periparturient care a) yes b)no

4.3.1. If yes, what kind of management? ………………

4.3.2. Calving facilities a) calving pen b) the same barn

4.3.3. Bedding in maternity area a) yes b)no

4.3.3.1. if yes, type of bedding a)stalks b) straw

4.3.4. Calving assistance a) routinely b) rarely c) never

4.3.4.1. When do you provide calving assistance?.......................................

4.3.5. At what time do you separate the calf from his dam………………………….

4.3.6. Navel treatment a) practiced b) not practiced

If practiced, type of treatment/chemical used…………………………….

4.4. Awareness about the importance of colostum to neonatal calves a) yes b) no

4.4.1. Do you feed colostrum to your calves a) yes b)no

4.4.2. If yes, a) partial colostrum b) complete colostrum

4.4.3. Method of feeding a) suckling b) hand feeding

4.4.4. Time of first colostrum feeding a) with in 6 hour b) 6-24 hour c) > 24 hours

4.4.5. Duration of feeding a) for 24 house b) 24 hour-4 days c) > 4 days

4.4.6. If hand feeding, source of feeding a) dam b) another cow c) both

4.5. Feeding and watering management

4.5.1. Type of feed a) milk b) milk replacer

4.5.2. Amount of milk/milk replacer given

a) Known, amount (Lt)……………… b) unknown, residual milk

4.5.3. Frequency of milk feeding/day a) once a day b) twice a day c)…………

4.5.4. Time (in days) of introducing feed other than milk or milk replacer ……………..

4.5.5. Mode of feeding a) free grazing b) stall feeding c) partial grazing

4.5.5.1. If free grazing, time (hr) of grazing………………..

4.5.6. Could you mention major types for each of the following classes of feeds you are

using for dairy cows and calves?

| Classes of feeds | a)yes b)no | If yes, type of feed |
| --- | --- | --- |
| 1. Improved forages |  |  |
| 2. Crop residues |  |  |
| 3. Concentrates |  |  |
| 4. If others (specify) |  |  |

4.6. Housing management

4.6.1. Housing type a) indoor b) outdoor/hutch

4.6.2 .Location of the calf pen a) In cow shed b) in separate pen/shed (cubicle)

4.6.3 If separate pen a) individual pen b) group pen

4.6.4 If group pen, number of calves kept /pen………….

4.6.5. Bedding in calf house a) present b) absent

4.6.6. If present what is the bedding material a) straw b)stalk

4.6.7. Frequency of calf pen cleaning a) every calf entry b) daily c) twice a day

4.6.8. Which group of calves are often receive better managerial attention?

a) male calves b) female calves c) both If male/female…………

4.6.9. Weaning age (m) a) local…………..b) cross…..

*5. Calf morbidity and mortality data*

5.2. Is calf mortality the problem of your farm? a) yes b) no

5.3. Total number of calves (<1yr) the farm lost during the last one year:

Local……Cross…………Total………

5.4. At which age group mortality was higher?

a) perinatal age (until 24 h after birth)

b) neonatal age (death between 1 and 28 d of age

c) older age (death between 1 and 6 month of age)

5.5. Diseases which are responsible for sickness and death of calves in order of importance.

a).…………..…b)……………..…..c)……….……….d)……………..…..e)………………

5.6. Which breeds of calves are highly susceptible to diseases? a) local b) cross

5.7. Measures taken to prevent disease problems…………………………..…………

5.8 .Calf weaning practices

5.9.1. Who weans the calf mostly?

a) The cow refusal c) Owner d) Refusal of the calf e) Others

*6. Dairy cow health disorders and health management activities*

6.1. Major Dairy cow health disorders……………………..

6.2. Pregnant cow vaccination a) yes b) no

6.3. Dry cow therapy a) yes b) no

*7. Milk production, marketing and constraints*

7.1. Current total milk produced/day (Lt)………Sold/day……...current price/liter…………

7.2. Mode of milk selling a) to milk cooperatives b) to local retailers c) direct selling

d) house hold consumption

7.3. Do you have an access to veterinary service? a) yes b) no

distance from home (Kms)…………

7.4. Could you mention major constraints of dairy production………………………

Annex II. Calf level data recording off sheet associated with dairy calf morbidity and mortality in Bahir Dar Milk-shed

Name of the owner…………………….Woreda…………..kebele……..…Tel….……….

Calf ID……………………..Dam ID……….………………….

*I****.*** *Calf and management associated factors*

1. Date of birth date…………………month……..…year….….…...

2. Condition of birth a) Easy b) Dystocia/assisted

2.1. If assisted, who assist the calving process? a) owner b) Veterinarian

3 Time of birth a) night b) day

4. Site of birth a) indoor/cow’s barn b)outdoor /field

5. Sex a) male b) female

6. Breed a) local b) cross

7. If cross, exotic blood level a) <=50% b) 50-75% c)>=75%

8. Navel disinfection a) yes b) no If yes, chemical used…………

9. The calf fed with maternal colostrum a) yes b) no

If no, why…………………………………………..

10. If yes, time of colostrum ingestion a) before 6 hr b) 6-12hr c) 12-24 d) >24hr

11. Method of colostrum feeding a) suckling b) hand feeding/bucket c) both

11.1. If hand fed, amount given……………..

11.2. Source of colostrum a) dam b) another cow c)both

11.3. Was the dam presented to the calf during hand feeding a) yes b)no

12. Vigor status as soon as birth

a) good vigor/quick suckling

b) poor vigor/ delayed suckling

13. Time of separation of the calf from dam/postpartum hr ?

a ) before 1^st^ nursing b) after 1^st^ nursing c)before 24 hr age d)after 24 hr age

14. Birth weight: Kg.……….., Weaning weight: Kg…………

15. Weaning age (m)………………………

*II. Dam associated factors*

16. Mothering instinct a) good mothering b) poor mothering

17. Parity of the dam a) primiparous/first b) multiparous/second and above

18. Dam breed a) local b) cross

19. If cross, exotic blood level a) <=50% b) 50-75% c)>=75%

20. Dam age………………………..

21. Lactation length (LL)…………Length of dry period (days)………..Open days……………

22. Age at first calving ……………Calving interval…

23. Milk yield(L/day): Early….................mid……..…..……late……..……

24. Other dam health disorders………………………………………………..

*III. Sire associated factors*

25. Source of breeding service a) AI b) natural mating/bull service

25.1. If AI, source of semen, a) H. fresian b) Jerssy c)…………………..

25.2. If natural mating, source of bull

a) home breed b) neighboring bull c) community bull

25.3. If home breed, is the bull relative to the calf? a) yes b) no

25.4. If yes, degree of relationship a) full sibs brother b) half sibs brother c) others

*VI. Calf case incidence record*

26. Date of appearance of clinical signs………………………….……….

27. Major clinical signs……………………………………………….……

28. Diagnosis……………………………… Treatment……………………

29. Treatment out come a) recovered b) died

If died, date of death………………………………………….…

Annex III. Standardized case definitions used during recording of diseases and mortality events between birth and 6 months/180 days of age in Bahir Dar milk-shed

| Disease condition | Case definition |
| --- | --- |
| Diarrhea/scours | Manure is of looser consistency than normal calves. Any condition characterized by passing of lose or watery feces with increased frequency, which could or could not be accompanied by other systemic signs like dehydration, decreased appetite or fever |
| Respiratory disease | Increased resting respiratory rate, fever (>39.5^0^c) with one or more additional signs such as coughing, nasal discharge, depression, decreased appetite or rough hair coat |
| Naval ill/Omphalitis | Warm enlargement of umblical cord, or foul smelling discharge from the umblical structures due to infection |
| Septicaemic cond. | Any condition characterized by depression, anorexia and fever without any distinct involvement of specific body system |
| LSD  Rabies | Characterized by skin nodules, fever, necrotic plaques in mucosae and lymphadenopathy.  A history of rabid dog bite preceded nervous signs like drooling of saliva, aggressiveness and beat their heads by any inanimate objects, extended recumbence and complete loss of appetite then ended up with death |
| Congenital problems | Any problems that was acquired inborn. |
| Miscellaneous cases | Different health problems that could not be grouped in any one of the other groups mentioned before and diagnosed relatively less frequently (traumatic injury, birth defects, ring worm, warts e.t.c…) |
| Perinatal mortality | Live-births that died until 24 hr of birth of life without an obvious disease |
| Neonatal mortality | Death between 1 and 28 days of age |
| Older calf mortality | Death between 1 and 6 month of age |

Source: (Heinriches and radostitis, 2001; Wudu, 2004; Windyer et al., 2014)

Annex IVV. Potential predictors variables considered for morbidity and mortality analysis in Bahir Dar milk-shed, Northwest Ethiopia

| **S/no** | **Variables** | **Description of category and codes** |
| --- | --- | --- |
| 1 | Calf Breed | 0 = cross  1 = local |
| 2 | Calf Age | 0 = <3 month of age (younger)  1 = ≥3 months of age (older) |
| 3 | Sex of calf | 0=male  1=female |
| 4 | Calf vigor status at birth | 0=good  1=poor |
| 5 | Ease of birth | 0=normal  1=dystocia |
| 6 | Calving site | 0=outdoor  1=barn |
| 7 | Birth time | 0=night  1=day |
| 8 | Parity of the dam | 0=primiparous  1=multiparous |
| 9 | Birth related disorder | 0=no  1=yes |
| 10 | Disease history during gestation | 0=no  1=yes |
| 11 | Birth type | 0=single  1=twin |
| 12 | Colostrum feeding | 0=unfed  1=fed |
| 13 | Calf housing | 0=Indoor  1=Outdoor |
| 14 | Method of colostrum feeding | 0 = suckling  1 = hand feeding |
| 15 | Calf accommodation | 0 = separate  1 = Group housing |
| 16 | Calf barn hygiene | 0=Clean  1=Unclean |
| 17 | Breeding method | 0=bull service  1=Artificial Insemination (AI) |
| 18 | Bedding | 0=no  1=yes |
| 19 | Age of the dairy farm | 0 = <5year  1 =>≥5 year |
| 20 | Dairy as a primary source of income | 0 = yes  1 = no |
| 21 | Calf care taker | 0 = owner  1= hired |
| 22 | Sex of calf caretaker | 0 = male  1 = female |
| 23 | Experience calf caretaker | 0 = ≤ 5 years  1 = >5 years |
| 24 | Total herd size | 0=<20 (Smallholder)  1=>20 (Large/specialized) |
| 25 | Study location/district | 0=Bahir Dar City  1=Bahir Dar zuria  2=Mecha  3=Yilmana Densa |
| 26 | Type of agriculture | 0=specialized livestock  1=mixed crop-livestock |
| 27 | Dairy production system | 0=urban  1=peri-urban |
| 28 | Altitude (m.a.s.l) | 0=<2000  1=≥2000 |
| 29 | Dam vaccination history | 0=unvaccinated  1=vaccinated |
| 30 | Mode of dairy feeding | 0=free grazing  1=stall feeding |
| 31 | Provision of improved forage | 0=no  1=yes |
| 32 | Provision of concentrate feed | 0=no  1=yes |
| 33 | Amount of milk given | 0=unknown/residual milk  1=known quantity |
| 34 | Water access | 0=limited  1=free access |
| 35 | Barn floor material | 0=dirt  1=cemented |
